# Evaluation on equality and efficiency of health resources allocation and health services utilization in China

**DOI:** 10.1186/s12939-017-0614-y

**Published:** 2017-07-14

**Authors:** Jian Sun, Hongye Luo

**Affiliations:** 1 School of Humanities and Social Science, Guangxi Medical University, 22 Shuang Yong Road, Qing Xiu District, Nanning, Guangxi Zhuang Autonomous Region 530021 China; 2 School of Information and Management, Guangxi Medical University, 22 Shuang Yong Road, Qing Xiu District, Nanning, Guangxi Zhuang Autonomous Region 530021 China

**Keywords:** Equality, Efficiency, Health resources allocation, Health services utilization, China

## Abstract

**Background:**

China is faced with a daunting challenge to equality and efficiency in health resources allocation and health services utilization in the context of rapid economic growth. This study sought to evaluate the equality and efficiency of health resources allocation and health services utilization in China.

**Methods:**

Demographic, economic, and geographic area data was sourced from China Statistical Yearbook 2012–2016. Data related to health resources and health services was obtained from China Health Statistics Yearbook 2012–2016. Furthermore, we evaluated the equality of health resources allocation based on Gini coefficient. Concentration index was used to measure the equality in utilization of health services. Data envelopment analysis (DEA) was employed to assess the efficiency of health resources allocation.

**Results:**

From 2011 to 2015, the Gini coefficients for health resources by population ranged between 0.0644 and 0.1879, while the Gini coefficients for the resources by geographic area ranged from 0.6136 to 0.6568. Meanwhile, the concentration index values for health services utilization ranged from −0.0392 to 0.2110. Moreover, in 2015, 10 provinces (32.26%) were relatively efficient in terms of health resources allocation, while 7 provinces (22.58%) and 14 provinces (45.16%) were weakly efficient and inefficient, respectively.

**Conclusions:**

There exist distinct regional disparities in the distribution of health resources in China, which are mainly reflected in the geographic distribution of health resources. Furthermore, the people living in the eastern developed areas are more likely to use outpatient care, while the people living in western underdeveloped areas are more likely to use inpatient care. Moreover, the efficiency of health resources allocation in 21 provinces (67.74%) of China was low and needs to be improved. Thus, the government should pay more attention to the equality based on geographic area, guide patients to choose medical treatment rationally, and optimize the resource investments for different provinces.

## Background

Equality and efficiency in health resources allocation and health services utilization are important goals pursued by health policy makers and health systems [1]. Moreover, equitable and efficient allocation of health resources is one of the basic conditions to the sustainable development of health services. The degree of attention to the equality and efficiency of health resources allocation and health services utilization issues continues to improve, but very few researchers have been undertaken into regional disparities and inefficiency of health resources allocation and health services utilization in China [2–4].

China is faced with a daunting challenge to equality and efficiency in health resources allocation and health services utilization in the context of rapid economic growth [5]. Due to the differences in economic development among the eastern, central and western regions, the health resources allocation and health services utilization in China is inequitable and inefficient [3, 6]. Compared with the developed eastern region, the central and western regions are economically underdeveloped. The eastern region has adequate health resources, whilst the central and western regions lack high-quality health resources. Additionally, the high cost of medical care services hinders the access to health services for poorer populations. In this paper, Gini coefficient was employed to evaluate the equality of health resources allocation from 2011 to 2015, concentration index was used to measure the equality in utilization of health services at the same time, and data envelopment analysis (DEA) was used to assess the efficiency of health resources allocation in the 31 provinces of China (excluding Hong Kong, Macao, and Taiwan) in 2015. The results of this study could shed light on the future health resources allocation and health services development in China.

## Methods

### Data sources and statistical analysis

Demographic, economic, and geographic area data was sourced from China Statistical Yearbook 2012–2016 [7–11]. Data related to health resources and health services was obtained from China Health Statistics Yearbook 2012–2016 [12–16].

Microsoft Excel 2013 was employed to calculate the Gini coefficient as well as concentration index and draw figures, and DEAP (V2.1) was used to conduct data envelopment analysis.

### Gini coefficient

Because the Gini coefficient has been identified as superior tool for evaluating the equality of health resources allocation [17], we employed it to examine the equality of health resources allocation (including health care institutions, health care beds and health workers) among provinces. It is derived from the Lorenz curve, reflecting the ratio of the area between the curve and the diagonal line, to the whole area below the 45^。^ line. The following formula was employed to calculate the Gini coefficient:

$$ \mathrm{G}=\sum_{i=1}^n\mathrm{Wi}\mathrm{Yi}+2\kern0.24em \sum_{i=1}^n\mathrm{Wi}\left(1-\mathrm{Vi}\right)-1 $$ [18],

where W_i_ is the cumulative proportion of the population or geographic area in each group; Y_i_ is the cumulative proportion of the health resources in each group; V_i_ = Y_1_ + Y_2_ + ……Y_i_; i is the fractional rank in terms of per capita health resources from the lowest number to the highest number.

The Gini coefficient ranges from 0 to 1; higher Gini coefficient indicates greater inequalities; a value of less than 0.2 suggests low inequality; a value of between 0.2 and 0.3 suggests moderate inequality; a value of between 0.3 and 0.4 suggests high inequality; a value of higher than 0.4 indicates extreme inequality [17, 19–26].

### Concentration index

As the concentration index has been identified as superior tool to measure the equality of health services utilization [17], we employed it to measure the equality of health services utilization (including outpatient visits, inpatients visits, and bed utilization rate) among provinces. We employed the following formula to calculate the concentration index:

$$ \mathrm{S}=\frac{1}{2}\sum_{\mathrm{i}=0}^{\mathrm{n}-1}\left(\mathrm{Yi}+\mathrm{Yi}+1\right)\left(\mathrm{Xi}+1-\mathrm{Xi}\right) $$

$$ \mathrm{CI}=2\times \left(0.5-\mathrm{S}\right), $$

where Y_0_ is 0 and X_0_ is 0; Y_i_ is the cumulative proportion of health services in each group, X_i_ is the cumulative proportion of population in each group, and i is the fractional rank according to per capita GDP beginning with the lowest; CI represents the concentration index [27, 28].

The concentration index ranges from −1 to +1; the greater the absolute value of concentration index, the greater the degree of inequalities; a value of 0 indicates absolute equality; a negative value indicates a concentration of the health service on the poorer populations; a positive value indicates a concentration of the service on the richer populations [24].

### Data envelopment analysis

Data envelopment analysis (DEA), first developed by Charnes et al. in 1978, is a non-parametric mathematical programming methodology that has been widely used to measure the relative efficiency or performance of decision-making units (DMUs) [29, 30]. In this study, we considered every province as an analytical unit. The Charnes, Cooper, and Rhodes (CCR) model, as the model of DEA, assumes that production is constant return to scale (CRS), which means that an increase in the inputs will lead to a proportionate increase in the outputs, and measure the overall efficiency (OE) and slack variables of each province [29]. The slack variable refers to the slack values of each province in terms of health resources allocation [29]. When the OE is 1, and all the slack variables are 0, the province is said to be relatively efficient [31]. When the technical efficiency (TE) is 1, and the scale efficiency (SE) and OE are less than 1, and all the slack variables are 0, the province is weakly efficient [32]. When the OE ranges from 0 to 1, and not all the slack variables are 0, the province is inefficient [32]. Moreover, the Banker, Charnes, Cooper (BCC) model assumes that the production is variable return to scale (VRS), which indicates that an increase in the inputs will lead to either an increase or a decrease in the outputs, and divide the OE into the TE and SE of each province [33–37].

$$ \mathrm{OE}={\mathrm{TE}}^{\ast }\ \mathrm{SE} $$

The CCR model and BCC model out of DEA models have been identified as superior tools for measuring the relative efficiency of health resources allocation [19], so we employed them to evaluate the relative efficiency of health resources allocation in the 31 provinces of China in 2015. The number of health care institutions, the number of health care beds and the number of health workers were selected as inputs; the number of outpatient visits, the number of inpatient visits and bed utilization rate were selected as outputs.

## Results

### Health resources and health services in China from 2011 to 2015

Table 1 showed the health resources and health services in China from 2011 to 2015. Totally, the number of the three health resources had been increasing from 2011 to 2015. Both the number of outpatient visits and the number of inpatient visits were increased at the same time, whereas the bed utilization rate decreased from 88.5% in 2011 to 85.4% in 2015.

**Table 1:** Health resources and health services in China from 2011 to 2015

Year	Input			Output		
	Health care institutions (unit)	Health care beds (unit)	Health workers (individuals)	Outpatient visits (times)	Inpatient visits (times)	Bed utilization rate(%)
2011	954,389	5,159,889	8,606,040	2,258,837,284	152,976,533	88.5
2012	950,397	5,724,775	9,108,705	2,541,616,095	178,570,984	90.1
2013	974,398	6,181,891	9,780,483	2,741,776,872	192,154,557	89.0
2014	981,432	6,601,214	10,224,213	2,972,069,922	204,411,818	88.0
2015	983,528	7,015,214	10,683,881	3,083,640,862	210,537,715	85.4

### Equality in the distribution of health resources

In order to evaluate the equality of health resources allocation comprehensively, we calculated the Gini coefficients both based on population and geographic area. The Gini coefficient by population means that its corresponding Lorenz curve’s x-axis is the cumulative proportion of population in each group, indicating the equality status based on population; while the Gini coefficient by geographic area means that its corresponding Lorenz curve’s x-axis is the cumulative proportion of geographic area in each group, which suggests the equality status based on geographic area.

Table 2 compared the Gini coefficients for health resources in China from 2011 to 2015. The Gini coefficients by population ranged between 0.0644 and 0.1879: 0.1845–0.1879 for the number of health care institutions, 0.0674–0.0739 for the number of health care beds, 0.0644–0.0752 for the number of health workers, which means that the distribution of the health resources shows a low level of inequality. Moreover, the Gini coefficients by geographic area ranged between 0.6136 and 0.6568: 0.6136–0.6177 for the number of health care institutions, 0.6366–0.6402 for the number of health care beds, 0.6553–0.6568 for the number of health workers, indicating that the distribution of the resources exhibits an extreme level of inequality.

**Table 2:** Gini coefficients for health resources in China from 2011 to 2015

Gini coefficient	Year	Health care institution	Health care bed	Health worker
Population size	2011	0.1879(low inequality)	0.0739(low inequality)	0.0752(low inequality)
	2012	0.1869(low inequality)	0.0708(low inequality)	0.0716(low inequality)
	2013	0.1859(low inequality)	0.0674(low inequality)	0.0686(low inequality)
	2014	0.1860(low inequality)	0.0685(low inequality)	0.0658(low inequality)
	2015	0.1845(low inequality)	0.0693 (low inequality)	0.0644(low inequality)
Geographic size	2011	0.6177(extreme inequality)	0.6398(extreme inequality)	0.6563(extreme inequality)
	2012	0.6136(extreme inequality)	0.6402(extreme inequality)	0.6568(extreme inequality)
	2013	0.6152(extreme inequality)	0.6392(extreme inequality)	0.6563(extreme inequality)
	2014	0.6145(extreme inequality)	0.6366(extreme inequality)	0.6553(extreme inequality)
	2015	0.6154(extreme inequality)	0.6390(extreme inequality)	0.6556(extreme inequality)

**Fig. 1:**
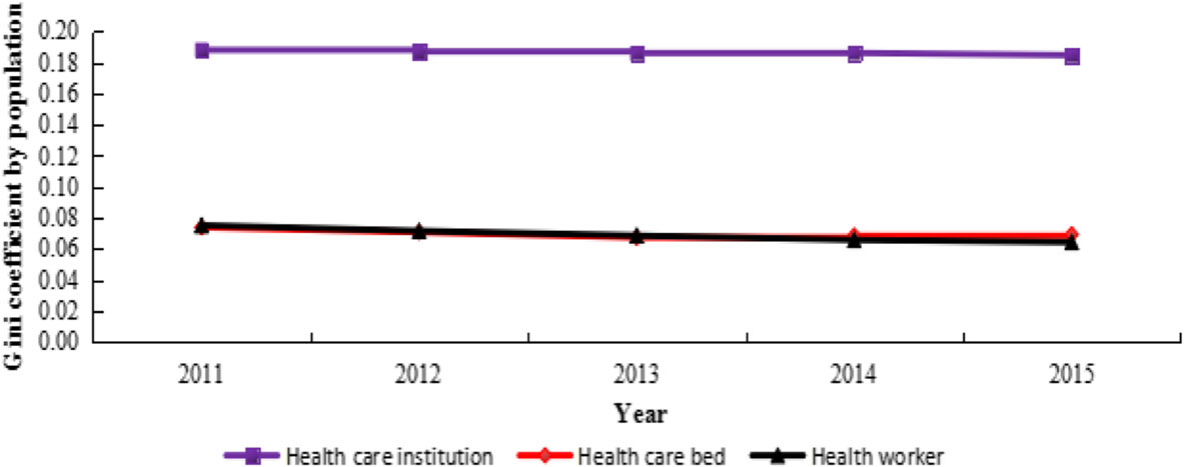
Gini coefficients for health resources by population in China from 2011 to 2015

**Fig. 2:**
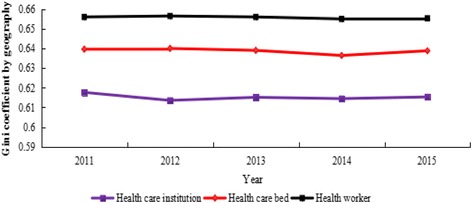
Gini coefficients for health resources by geographic area in China from 2011 to 2015

Figure 1 showed the Gini coefficients for health resources by population from 2011 to 2015. Figure 2 showed the Gini coefficients for the resources by geographic area in the meanwhile. The Gini coefficients both based on population and geographic area for the resources showed an overall downward trend, which indicates that the equality status got better.

### Equality in utilization of health services

Table 3 showed the concentration index values for health services utilization in China from 2011 to 2015. Meanwhile, the concentration index values for health services utilization ranged from −0.0073 to 0.2110. The concentration index values for outpatient visits, ranged from 0.1944 to 0.2110, suggesting a concentration of the service towards the richer populations. Conversely, the concentration index values for inpatients visits ranged from −0.0392 to −0.0126, and the concentration index values for bed utilization rate ranged from −0.0142 to −0.0073, indicating a concentration of these services towards the poorer populations.

**Table 3:** Concentration index values for health services utilization in China from 2011 to 2015

Year	Outpatient visits	Inpatients visits	Bed utilization rate
2011	0.2110	−0.0285	−0.0130
2012	0.2035	−0.0392	−0.0142
2013	0.2033	−0.0126	−0.0126
2014	0.1975	−0.0260	−0.0083
2015	0.1944	−0.0223	−0.0073

**Fig. 3:**
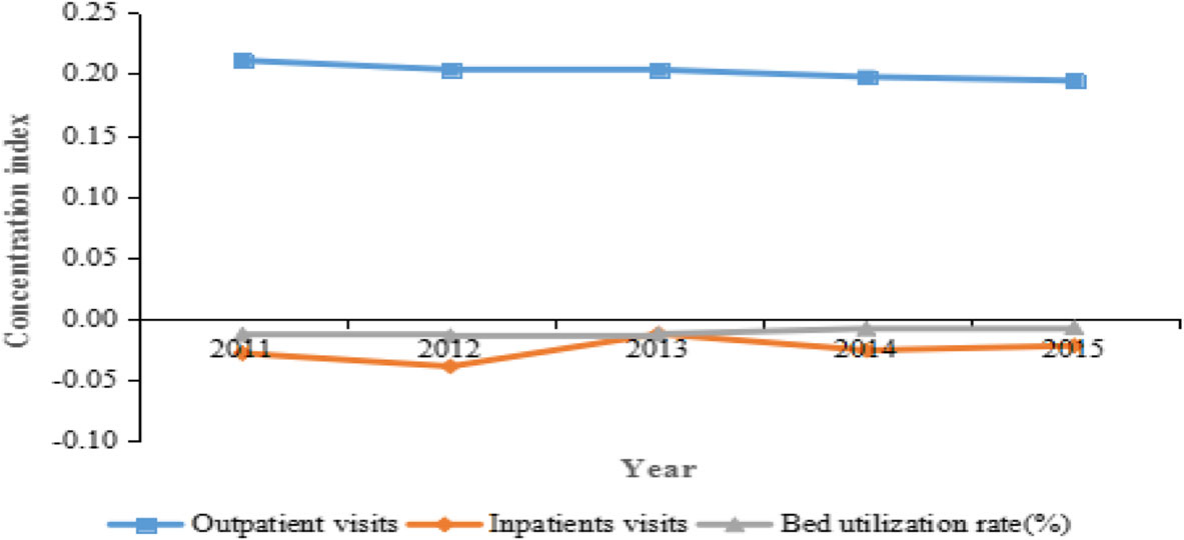
Concentration index values for health services utilization in China from 2011 to 2015

Figure 3 showed the concentration index values for health services utilization in China from 2011 to 2015. In the meanwhile, the concentration index values for outpatient visits showed an overall downward trend, while the concentration index values for inpatients visits, bed utilization rate showed an overall upward trend, which indicated that the equality status of these services utilization got better. Meanwhile, the absolute values of concentration index for outpatient visits were significantly higher than those of other services, indicating that the equality status was the worst. On the contrary, the absolute values of concentration index for bed utilization rate were lower than those of other services, which indicates that the equality status was the best.

### Efficiency evaluation based on data envelopment analysis

#### Operational efficiency analysis

Table 4 showed the efficiency values and slack values in health resources allocation of the 31 provinces in China in 2015. According to the scores, the average scores of OE, TE, and SE in the 31 provinces were 0.904, 0.921 and 0.982, respectively. Among the 31 provinces, 10 provinces (32.26%), such as Beijing, Tianjin, and Shanghai, had efficiency scores of 1 for OE, TE, and SE, and all the slack variables were 0, indicating that health resources allocation in these provinces was relatively efficient. Furthermore, 7 provinces (22.58%), such as Jiangsu, Zhejiang, and Anhui, had TE scores of 1, OE and SE scores of less than 1, a slack variable of 0, thus health resources allocation in these provinces was weakly efficient. Finally, 14 provinces (45.16%), such as Hebei, Shanxi, and Inner Mongolia, had OE, TE and SE scores of less than 1, and not all the slack variables were 0, thus they were inefficient in terms of health resources allocation. Among the 14 inefficient provinces, Shanxi had the lowest OE score of 0.588, suggesting that its efficiency was 58.80% of that of the efficient provinces. Of the 20 scale-inefficient provinces, 9 provinces (45.00%), such as Shanxi, Inner Mongolia, and Jilin, had increasing return to scale (IRS), suggesting that these scale-inefficient provinces had scales that were too small, and they had to expand their scale of operation; the remaining 11 provinces (55.00%), such as Hebei, Liaoning, and Jiangsu, had decreasing return to scale (DRS), indicating that these scale-inefficient provinces had to cut down their operations to achieve CRS.

**Table 4:** Efficiency values and slack values in the 31 provinces of China in 2015

Provinces	Overall efficiency	Technical efficiency	scale efficiency	Type of scale efficiency	S^1−^	S^2−^	S^3−^	S^1+^	S^2+^	S^3+^	Relatively efficiency status
Beijing	1.000	1.000	1.000	−	0	0	0	0	0	0	Efficient
Tianjin	1.000	1.000	1.000	−	0	0	0	0	0	0	Efficient
Hebei	0.806	0.817	0.986	DRS	37,342	62,436	97,330	0	0	4.378	Inefficient
Shanxi	0.588	0.591	0.994	IRS	26,227	74,891	120,527	0	0	9.331	Inefficient
Inner Mongolia	0.647	0.653	0.990	IRS	12,732	46,397	73,638	0	0	12.519	Inefficient
Liaoning	0.762	0.775	0.984	DRS	11,938	66,044	78,509	0	0	3.887	Inefficient
Jilin	0.706	0.709	0.995	IRS	8444	42,010	62,271	0	0	7.537	Inefficient
Heilongjiang	0.759	0.761	0.998	IRS	4965	53,164	68,410	18,611,938	0	7.441	Inefficient
Shanghai	1.000	1.000	1.000	−	0	0	0	0	0	0	Efficient
Jiangsu	0.967	1.000	0.967	DRS	0	0	0	0	0	0	Weakly efficient
Zhejiang	0.943	1.000	0.943	DRS	0	0	0	0	0	0	Weakly efficient
Anhui	0.990	1.000	0.990	DRS	0	0	0	0	0	0	Weakly efficient
Fujian	0.882	0.885	0.997	IRS	10,158	19,895	32,351	0	0	7.816	Inefficient
Jiangxi	1.000	1.000	1.000	−	0	0	0	0	0	0	Efficient
Shandong	0.797	0.939	0.848	DRS	4696	43,217	180,217	18,959,586	0	3.852	Inefficient
Henan	0.843	0.953	0.884	DRS	4034	23,184	77,957	67,288,228	0	0	Inefficient
Hubei	0.979	1.000	0.979	DRS	0	0	0	0	0	0	Weakly efficient
Hunan	1.000	1.000	1.000	−	0	0	0	0	0	0	Efficient
Guangdong	1.000	1.000	1.000	−	0	0	0	0	0	0	Efficient
Guangxi	1.000	1.000	1.000	−	0	0	0	0	0	0	Efficient
Hainan	0.904	0.915	0.988	IRS	428	3285	15,726	1,167,618	0	3.761	Inefficient
Chongqing	1.000	1.000	1.000	−	0	0	0	0	0	0	Efficient
Sichuan	0.935	1.000	0.935	DRS	0	0	0	0	0	0	Weakly efficient
Guizhou	0.951	0.951	1.000	−	4273	9532	12,576	11,209,602	0	6.534	Inefficient
Yunnan	0.992	1.000	0.992	DRS	0	0	0	0	0	0	Weakly efficient
Tibet	1.000	1.000	1.000	−	0	0	0	0	0	0	Efficient
Shannxi	0.798	0.800	0.998	IRS	12,444	42,438	70,079	0	0	5.008	Inefficient
Gansu	0.815	0.817	0.997	IRS	14,526	23,418	33,263	0	0	3.031	Inefficient
Qinghai	0.958	0.988	0.970	IRS	1428	4712	581	3,718,718	0	5.194	Inefficient
Ningxia	1.000	1.000	1.000	−	0	0	0	0	0	0	Efficient
Xinjiang	0.995	1.000	0.995	DRS	0	0	0	0	0	0	Weakly efficient
Mean	0.904	0.921	0.982	/	4956	16,601	29,788	3,901,796	0	3	/

S^1−^, S^2−^, S^3−^, S^1+^, S^2+^, and S^3+^ represent the slack values of health care institutions, health care beds, health workers, outpatient visits, inpatient visits, and bed utilization rate, respectively

*Abbreviations*: *IRS* increasing return to scale, *DRS* decreasing return to scale. -: constant return to scale

#### Slack variable analysis

Compared with the efficient provinces, the inefficient provinces are supposed to either reduce their inputs or increase their outputs to improve the efficiency of health resources allocation. Aiming at achieving a relatively optimal output value, the inefficient provinces should reduce the average number of health care institutions by 7316, the average number of health care beds by 24,506, and the average number of health workers by 43,973 while maintaining their current output levels unchanged. Alternatively, the inefficient provinces ought to increase the average outpatient visits by 5,759,795 and increase the bed utilization rate by 4% at the current input levels.

## Discussion

From 2011 to 2015, the Gini coefficients for health care institution, health care bed, health worker by population were less than 0.2, indicating low inequality based on the above criteria. Conversely, the Gini coefficients for the three resources by geographic area exceeded 0.6 in the meanwhile, indicating extreme inequality. Obviously, the Gini coefficients by population are significantly lower than those by geographic area, indicating that there was a larger disparity in the geographic distribution of health resources than that in the population distribution, which was consistent with the finding of Jin et al. [38]. A potential explanation for this finding is that the government set the number of health resources per thousand population, rather than the number of health resources per 10,000 square km, as the allocation criterion [39]. As mentioned above, the equality status of the health resources allocation got better, which was consistent with the findings of Zhang et al. [40]. Consequently, it is reasonable to suggest the government that it should pay more attention to the equality based on geographic area when making regional health planning, perfect the allocation mechanism of health resources, and allocate more health resources to remote and economically underdeveloped provinces in order to improve the equality status of health resources allocation. Furthermore, the government ought to introduce adequate and experienced health workers in remote and economically underdeveloped provinces by giving extra subsidies and other preferential policies to ameliorate the inequality status of health worker.

The concentration index values for outpatient visits were positive, indicating that the people living in the eastern developed areas are more likely to use outpatient care than their western and central counterparts [17]. Conversely, the concentration index values for inpatients visits, bed utilization rate were negative, which indicates that the people living in western underdeveloped areas are more likely to use inpatient care than their eastern and central counterparts [17]. This disparity maybe due to the gaps in income level among the eastern, central and western regions [17]. Consequently, the government should pay attention to this phenomenon, guide patients to choose health services rationally, and control the rapid increase of health services prices.

More than 30% of provinces were relatively efficient in terms of health resources allocation. Moreover, approximately 22.58% of provinces were weakly efficient. That is to say, the scale of the existing health resources in these weakly efficient provinces was relatively smaller than others and the growth rate of outputs was higher than that in investment, thus more investment should be put to them. It is worth noting that more than 45% of provinces were relatively inefficient. In other words, the health resources in these provinces were not fully utilized at the current size. The results are consistent with Zhang’s study [41], which showed that 41.94% of the provinces in China were relatively inefficient in 2011. Thus, it is reasonable for these provinces to ameliorate their management strategies and improve the quality of health services to improve the efficiency of resource allocation.

This study has some limitations. On the one hand, the evaluation on equality and efficiency of health resources allocation and health services utilization was conducted independently in two phases, which needs to be improved in future studies. On the other hand, some representative indicators, such as the indicators related to health resources allocation were adequate; while some indicators, such as the benefits for the patients, for evaluating equality of health services were inadequate, which may have effects on the comprehensiveness of the evaluation.

## Conclusion

Based on the analysis above, we find that there exist distinct regional disparities in the distribution of health resources in China, which are mainly reflected in the geographic distribution of health resources. Furthermore, the people living in the eastern developed areas are more likely to use outpatient care, while the people living in western underdeveloped areas are more likely to use inpatient care. Moreover, the efficiency of health resources allocation in 21 provinces (67.74%) of China was low and needs to be improved. Consequently, stakeholders, including government, health care institutions, and patients, should cooperate jointly to improve the equality and efficiency of health resources allocation and health services utilization.

## Abbreviations

BCC model: Banker, Charnes, Cooper model
CCR model: Charnes, Cooper, Rhodes model
CRS: Constant return to scale
DEA: Data envelopment analysis
DMUs: Decision-making units
DRS: Decreasing return to scale
IRS: Increasing return to scale
OE: Overall efficiency
SE: Scale efficiency
TE: Technical efficiency
VRS: Variable return to scale
